# Topical betulinic acid for treatment of equine melanoma and sarcoid

**DOI:** 10.3389/fvets.2026.1821503

**Published:** 2026-05-28

**Authors:** Caitlin Moreno, Margaret Mudge, Rikki Horne, Jonathan Yardley

**Affiliations:** 1 Department of Veterinary Clinical Sciences, Ohio State University, Columbus, OH, United States; 2 The Ohio State University Veterinary Medical Center, Columbus, OH, United States

**Keywords:** betulinic acid, horse, melanoma, sarcoid, skin, topical

## Abstract

**Introduction:**

Betulinic acid, a natural pentacyclic triterpenoid, has demonstrated anti-inflammatory and anti-tumor properties in vitro, including activity against equine melanoma and sarcoid. However, there is limited in vivo evidence evaluating its clinical efficacy. The objective of this study was to assess the safety and efficacy of a compounded 1% betulinic acid cream for the treatment of equine cutaneous melanoma and sarcoid.

**Methods:**

This prospective, randomized, double-blinded, placebo-controlled clinical trial enrolled client-owned horses with melanoma or sarcoid tumors. Horses received either a compounded 1% betulinic acid cream or placebo cream, applied twice daily for 7 days followed by once daily for 21 days. Tumor measurements and photographs were obtained before and after the treatment period. Tumor volume was calculated using standard morphometric methods. Data were analyzed using a linear mixed-effects model with treatment group and tumor type as fixed effects and horse as a random effect. Owners recorded adverse events using daily questionnaires.

**Results:**

A total of 37 horses completed the study, including 16 with melanoma and 21 with sarcoid tumors. There were no statistically significant differences in tumor volume change between treatment and placebo groups for either tumor type. Tumor volume decreased in a proportion of cases across all groups. The treatment was well tolerated, with only mild, self-limiting local reactions reported.

**Discussion:**

Topical application of a compounded 1% betulinic acid cream was safe but did not result in a statistically significant reduction in tumor volume compared to placebo over a 30-day treatment period. These findings suggest that longer treatment durations or alternative dosing regimens may be necessary to achieve clinical efficacy. Further studies are warranted to evaluate extended treatment protocols and optimize therapeutic outcomes.

## Introduction

Melanoma and sarcoid are common equine tumors, representing approximately 4 and 50% of equine tumors, respectively. Historic data suggests approximately 80% of grey horses develop at least one melanotic tumor in their lifetime with more recent publications reporting 67% of grey horses having evidence of cutaneous melanoma on physical exam (1–3, 19, 20). Treatment of these tumors can be challenging and further limited based on size and location. Although there are topical treatments for equine sarcoid, there are little to no topical options for equine melanoma. Even those available for sarcoid, such as acyclovir, imiquimod, and *Sanguinaria canadensis* are used with variable success and can produce significant local reactions (4, 5). Betulinic Acid (BA) is a pentacyclic triterpenoid of plant origin, most notably from the white birch tree. It has been shown, *in vitro*, to induce apoptotic cell death in neoplastic cells through the mitochondrial (intrinsic) pathway while sparing normal cells and tissues (6). Betulinic acid has been studied in-vitro for canine lymphoma and osteosarcoma, demonstrating anti-tumor activity in a time and dose dependent manner (7). Recently, 1% BA and its derivative NVX-207 have been studied for their anti-tumor activity against equine melanoma (EM) and sarcoid (ES) and demonstrated efficacy in-vitro (8–11). Although an in-vivo study has been published evaluating the compound for treatment of melanoma, there are no prospective, blinded, placebo-controlled studies (12). Furthermore, to the authors’ knowledge, only in-vitro work is published regarding efficacy of the compound for equine sarcoid and there is no tolerability data available (9). Further research is needed to evaluate these compounds in clinical cases with comparison to a placebo group. Therefore, the objective of this study is to evaluate the safety and efficacy of a compounded 1% betulinic acid cream for the treatment of equine cutaneous melanoma and sarcoid in clinical cases. It was hypothesized that the treatment cream would be well tolerated at the tested concentration and would result in a reduction in tumor size over a 30-day period when compared to a placebo cream.

## Materials and methods

### Study design

This is a randomized, double-blinded, placebo controlled clinical trial. Horses were enrolled on a prospective basis and were recruited based on DVM referral or owner inquiry based on university advertising of clinical trial enrollment. Grey horses with melanotic masses described as darkly pigmented cutaneous masses in typical locations, such as perianal or tail base, did not have biopsies performed. In horses with numerous melanotic masses, those that were well demarcated, and solitary were selected for inclusion if possible. Horses in the sarcoid group were either enrolled based on the established classification scheme published for equine sarcoid or had biopsies performed to confirm the diagnosis (13). There were no breed or age restrictions. Photographs and measurements were obtained before and after the treatment period by a licensed veterinarian. The application of the cream (treatment or placebo) over the trial period was performed under field conditions by owner or caretaker.

### Randomization and blinding

A computer-generated random allocation sequence was created by the dispensing pharmacist. The pharmacist prepared identical study creams according to the allocation list and was the only individual aware of treatment assignment. Investigators and horse owners remained blinded throughout the study.

### Power analysis and outcome determination

An *a priori* power analysis was conducted to determine the minimum sample size required to detect a statistically significant difference in treatment response rates between the treatment and placebo groups. The calculation was based on a two-sided z-test for two independent proportions, with a significance level (*α*) set at 0.05 and a desired statistical power (1-*β*) of 80%. For the equine sarcoid group, the sample size calculation was based on previously published data for topical treatments, which report response rates between 75 and 85%. A conservative treatment response rate of 75% was used for this calculation. The spontaneous regression rate (placebo response) for sarcoids is low; this was estimated at 20%. Based on these parameters, a minimum of 13 horses per group would be required to detect a significant difference. For the equine melanoma group, no comparable efficacy data for topical treatments is available. Therefore, the sample size was calculated based on detecting a clinically meaningful treatment effect. A successful outcome was defined as achieving a 60% response rate in the treatment group, compared to a hypothetical baseline or placebo response rate of 10%. To detect this effect with 80% power, a minimum of 15 horses per group is required. To ensure the study is adequately powered for both conditions, the larger of the two calculated sample sizes will be adopted. Therefore, a minimum of 15 horses per group were recruited for both the sarcoid and melanoma groups.

### Topical cream composition and application protocol

**Figure 1:**
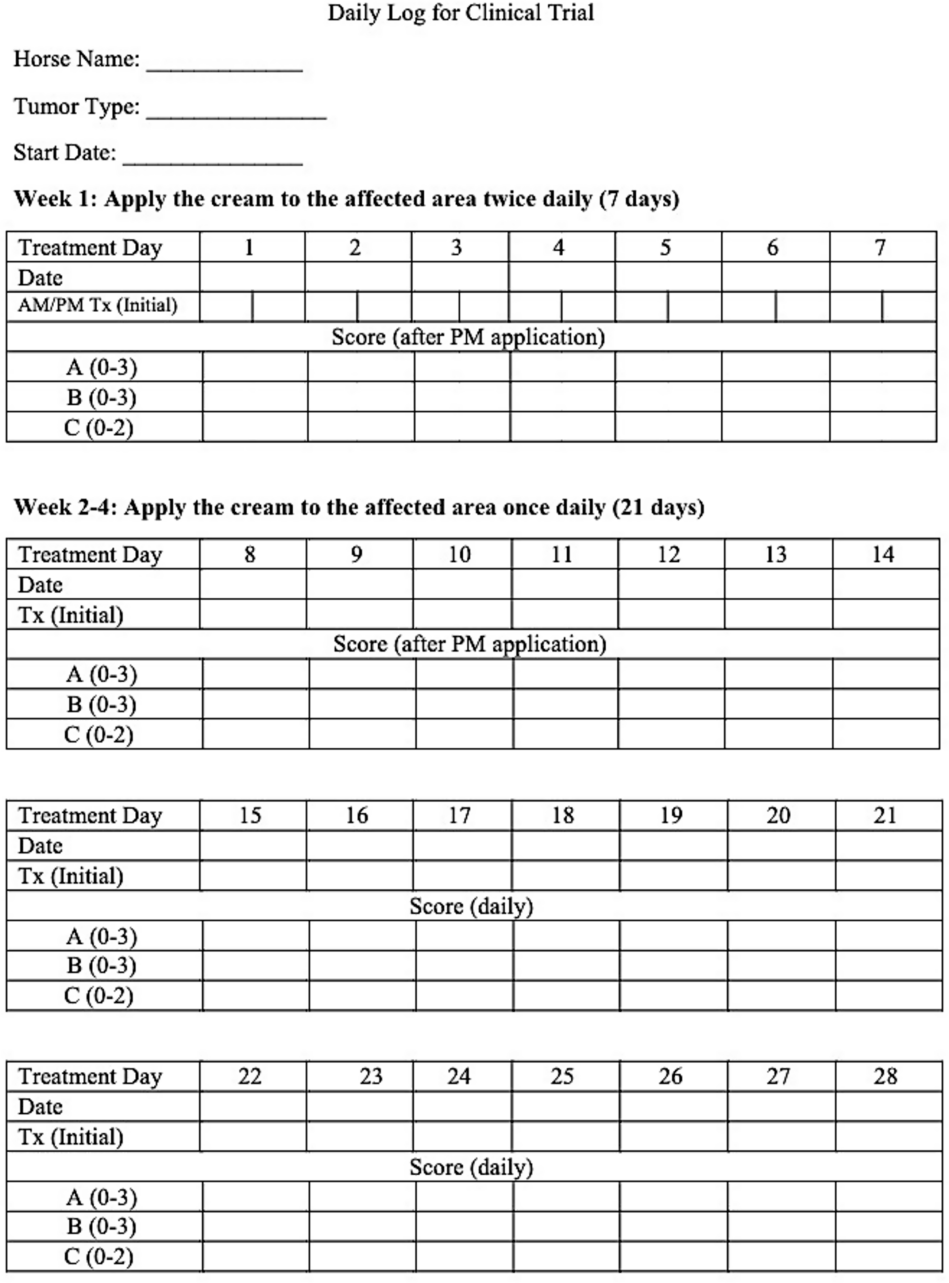
Daily log to track application compliance and tolerance.

**Figure 2:**
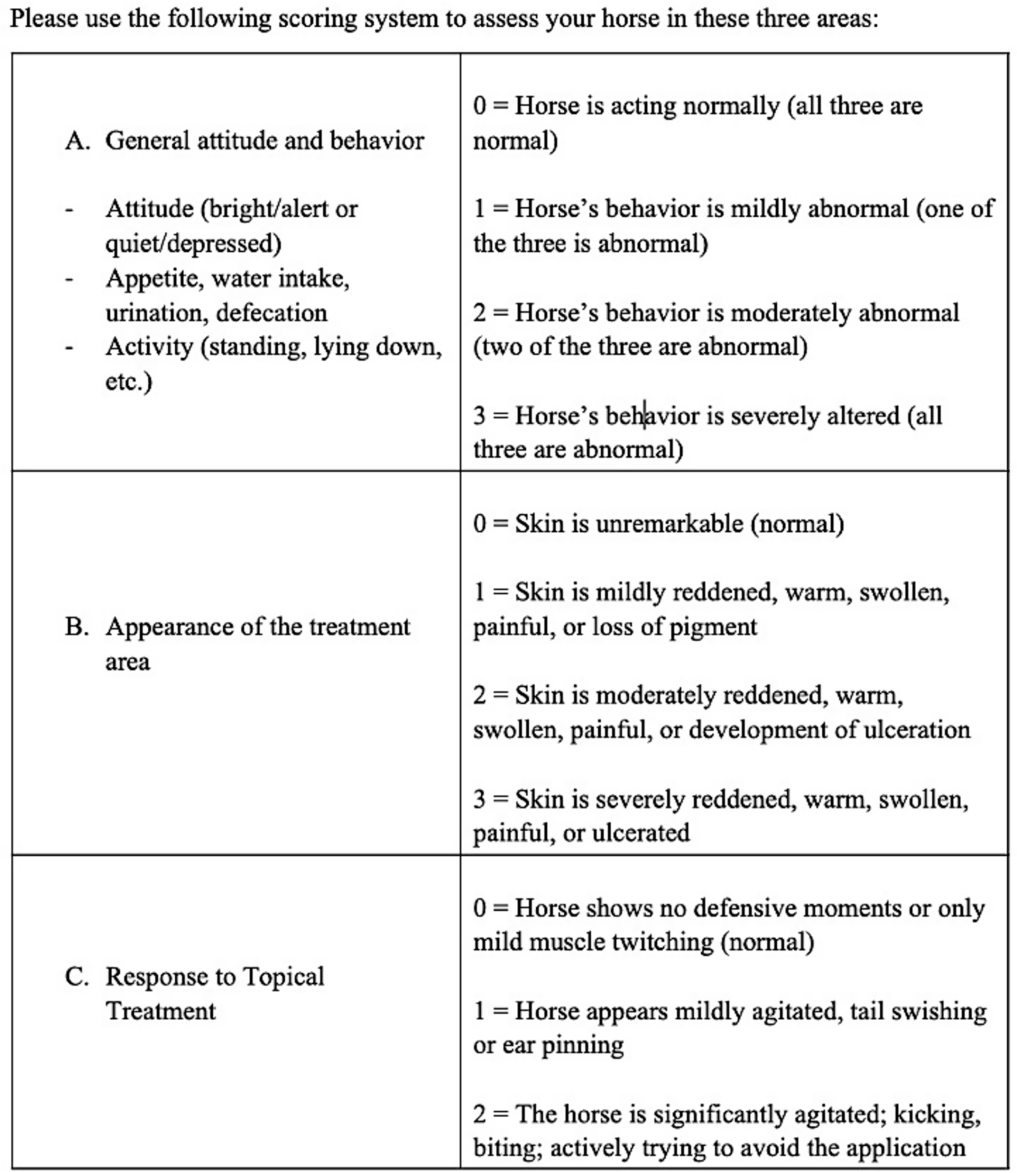
Daily questionnaire guidelines to assess tolerance to cream application.

The cream was made based on a previously reported formulation (12). The placebo cream was composed of Basiscreme DAC (Bombastus-Werke AG, Deutschland) with 20% parts Medium-Chain Triglyceride (MCT), NF oil (Spectrum Chemical MFG Corp, New Jersey). The treatment cream consisted of the same ingredients as the placebo, with the addition of 1% betulinic acid powder (Purity >98%) (Atkin Pharmaceuticals Incorporated, Chengdu, China). The creams were compounded the day of patient enrollment and given a beyond use date of 35 days. The treatment or placebo cream was applied twice daily for 7 days, then once daily for 21 days. The cream was applied with a gloved hand in a thin layer sufficient to cover the entire lesion and was refrigerated between applications to preserve potency. Owners were required to complete a daily treatment log (Figure 1) with the administering individual’s initials. They were also required to complete a questionnaire (Figure 2) daily to evaluate adverse reactions. This included three categories - general attitude and behavior (graded 0–3), appearance of the treatment area (graded 0–3), and the horse’s response to topical treatment (graded 0–2). After the application period, recheck photographs and measurements were obtained. The group was unblinded to the owner and clinician after analysis.

### Data analysis

Width and length measurements were obtained using calipers. Tumor volume was calculated based on an established model [V = w^2^ × (l/2)] for cutaneous masses (14, 15). To reduce measurement bias, a standardized measurement protocol was used and, whenever possible, measurements were performed by the same trained investigator. A clinically meaningful change in tumor volume was predefined as a reduction exceeding expected caliper measurement variability (≥20% from baseline). Data analysis was performed using statistical software (SPSS, Version 29, IBM). Tumor volume data was analyzed as a continuous outcome using a Linear Mixed-Effects Model (LMM) with treatment group and tumor type as fixed effects, including their interaction, and a random intercept for horse to account for repeated measurements within horses. Statistical significance was set at *p* ≤ 0.05. The information in the questionnaires was evaluated descriptively.

A *post-hoc* power analysis was conducted to confirm the statistical adequacy of the final sample sizes for both the sarcoid and melanoma cohorts, with a significance level (*α*) set at 0.05. A minimum detectable effect size analysis was performed. This analysis, which accounted for the clustered nature of melanoma tumors using a Generalized Linear Mixed-Effects Model (GLMM), determined the smallest effect the study could reliably detect with 80% power. Due to the unknown true value of the Intraclass Correlation Coefficient (ICC), a sensitivity analysis was performed across a range of plausible ICC values.

## Results

### Population

**Figure 3:**
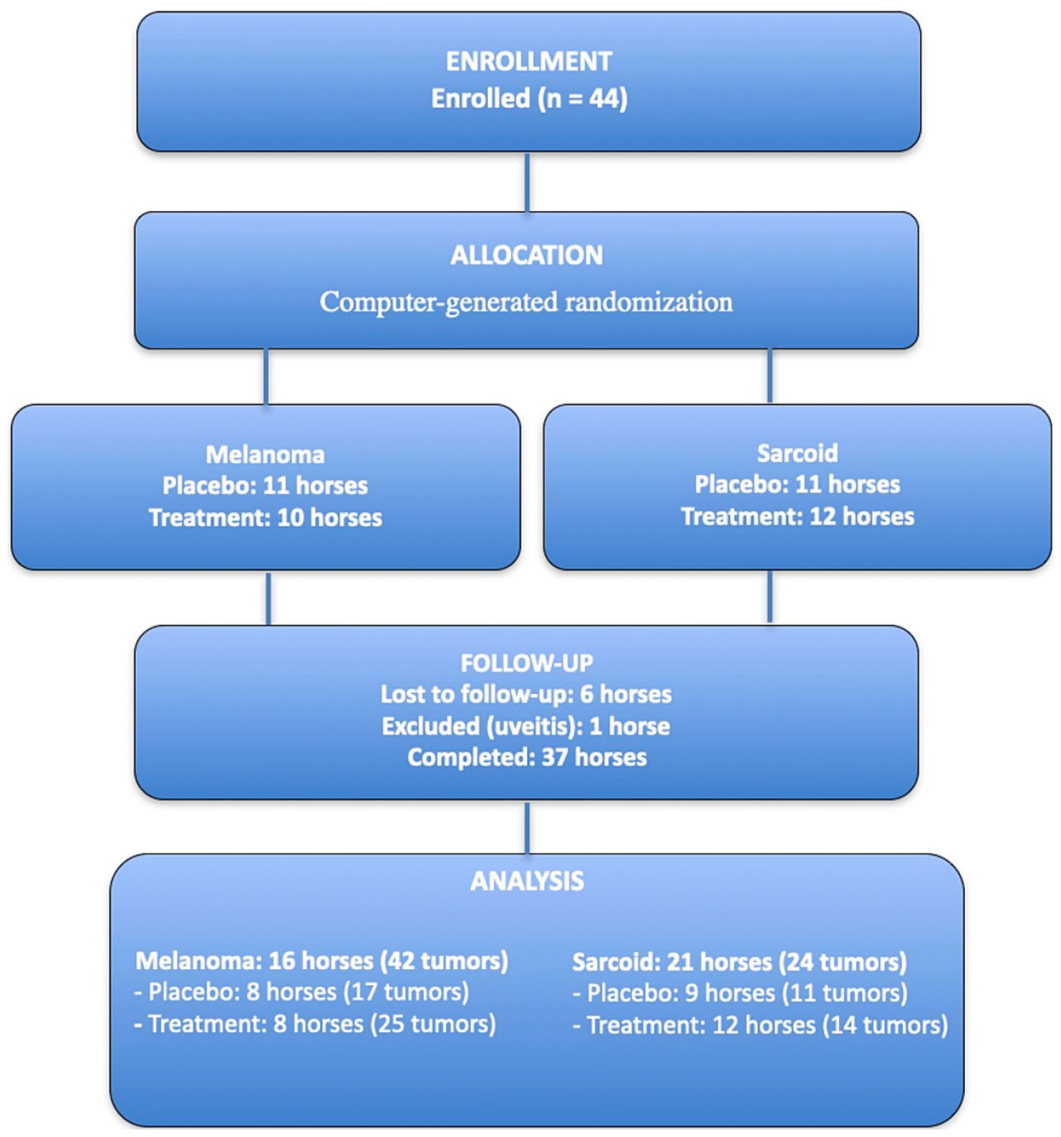
Flow diagram illustrating enrollment and allocation.

**Figure 4:**
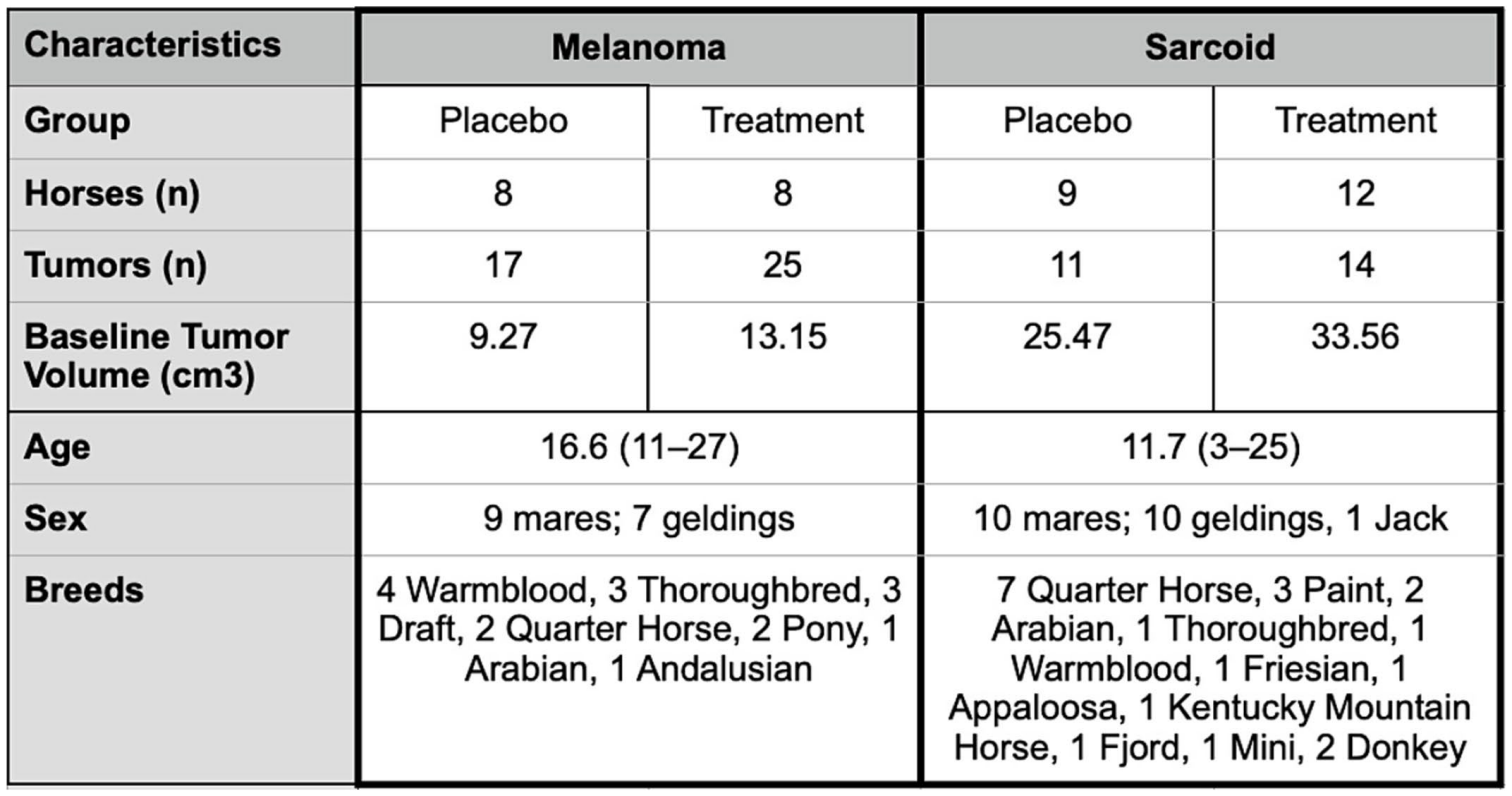
Baseline data for each group.

A total of 44 horses were enrolled (Figure 3). Six horses were lost to follow-up due to unsuccessful attempts to contact the owner for repeat measurements, and one was excused for unrelated health reasons necessitating other medications for management (historic uveitis). Out of the 37 horses that completed the study, there were 16 horses with melanoma, 42 tumors, and 21 horses with sarcoid, 24 tumors. The baseline characteristics of each group are presented in Figure 4. Tumors in the melanoma group were located under the tail, perineum (primarily), distal limb, inguinal region, and trunk. Of the 21 horses with sarcoid that completed the study, 11 had biopsies with a histopathologic diagnosis of sarcoid. Sarcoid lesions were mainly on the face (Periocular/muzzle/etc.), neck, and trunk.

For the sarcoid group (*n =* 21 horses), the sample was evaluated against the large treatment effect reported in the literature for similar therapies. This analysis confirmed that the study achieved very high statistical power (>90%) to detect an increase in tumor response rate from a 20% baseline to 75% in the treatment group. For the melanoma group (*n =* 16 horses with 42 tumors), a minimum detectable effect size analysis was performed. The results indicated that the study achieved a statistical power of approximately 85% under a low ICC of 0.1, and 71% under a moderate ICC of 0.3. This demonstrates that the study was sufficiently powered to detect a large treatment effect under realistic assumptions of low-to-moderate tumor correlation.

### Tumor data

**Figure 5:**
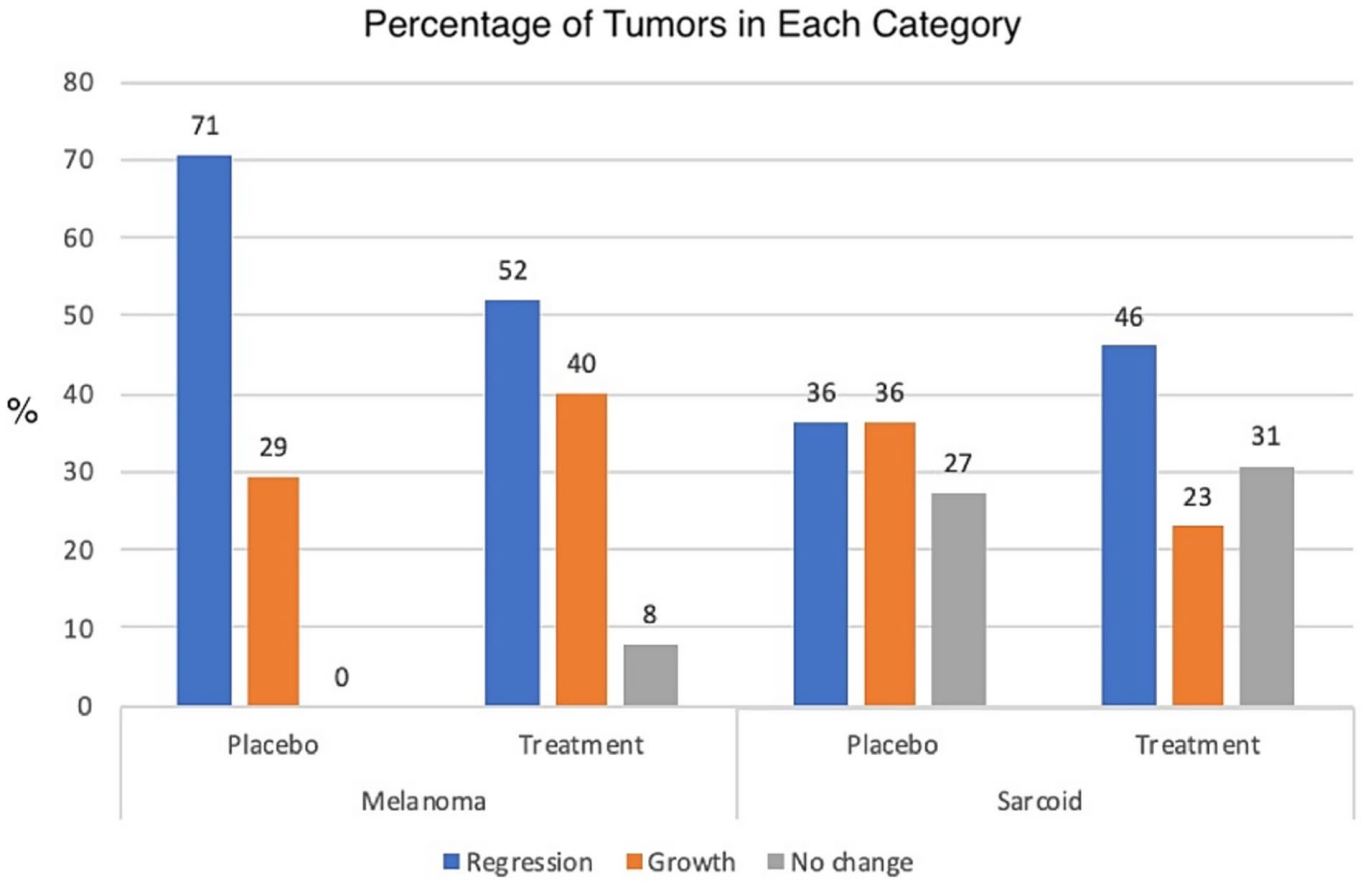
The percentage of tumors in each category based on the change in tumor volume over the 30 day trial period.

Baseline tumor volumes did not differ significantly between groups. The linear mixed-effects model demonstrated no significant effect of treatment group, tumor type, or their interaction on tumor volume over the study period. On average, tumor volume decreased for all groups. Sarcoid lesions in the treatment group tended to be larger by volume than those in the placebo group. Overall, 71% of the melanomas in the placebo group, 60% of melanomas in the treatment group, 63% of sarcoids in the placebo group, and 77% of sarcoid in the treatment group maintained the same tumor volume or decreased in tumor volume at the completion of the application period (Figure 5). Of the tumors that increased in volume (*n =* 22) over the study period, the average percent volume growth was 176% for the melanoma treatment group, 186% for the melanoma placebo group, 29% for the sarcoid treatment group, and 21% for the sarcoid placebo group. Biopsy of sarcoid tumors (*n =* 11) did not result in clinically relevant lesion deterioration.

### Tolerance data

**Figure 6:**
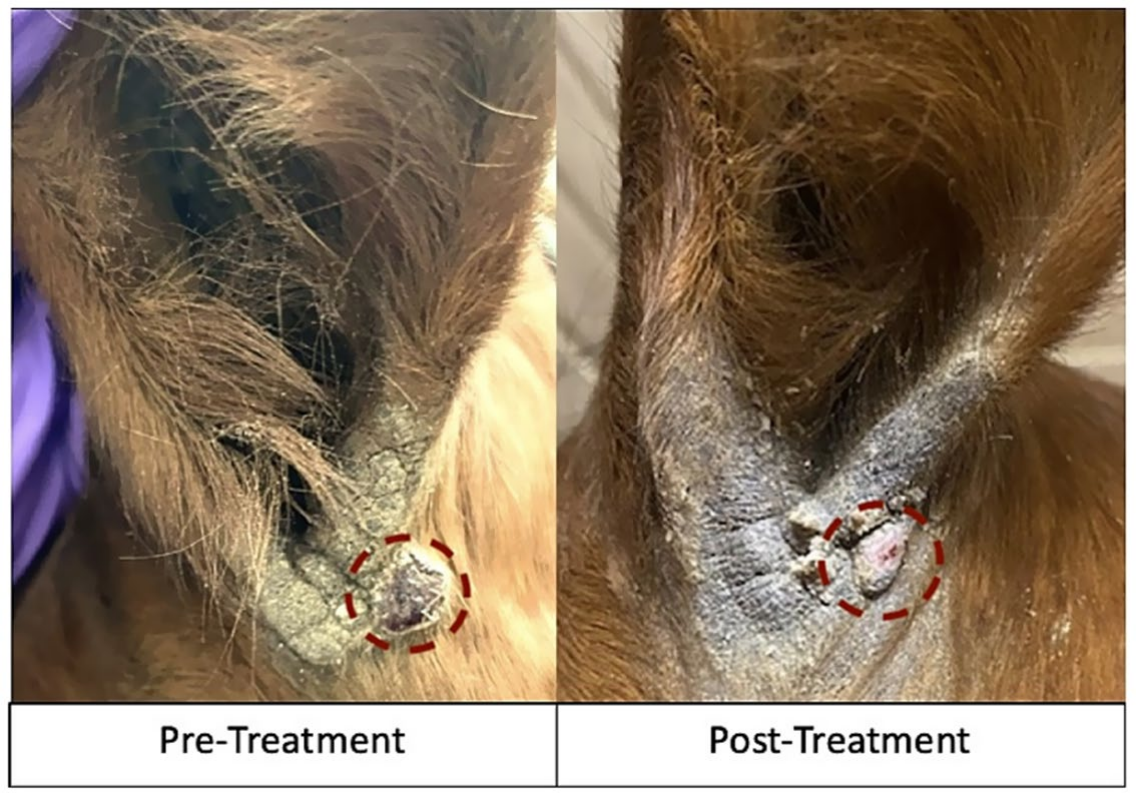
Image of ear sarcoid in treatment group pre- and post-treatment. The biopsy location is circled.

Local reactions were mild and self-limiting and did not affect continued application of the compound. For “general attitude and behavior,” no horses were scored above a 1, with the mode being 0. For “appearance of the treatment area’” no horses were scored above a 2, with the mode being 0. For “response to topical treatment,” no horses were scored above a 1, with the mode being 0. Some owners reported that the skin was subjectively smoother, and tumors appeared more rounded in some sarcoid cases in the treatment group (Figure 6).

## Discussion

This was the first prospective, double-blinded, placebo-controlled study to evaluate topical betulinic acid for equine melanoma and sarcoid, conditions for which current topical treatments can produce significant local inflammation (16). The primary finding was that a 30-day application of a 1% betulinic acid cream was safe and well-tolerated in a diverse clinical population but was not effective for significantly reducing melanoma tumor volume compared to placebo. These results provide a valuable foundation for future research and important clinical context for veterinarians considering this therapy.

The most critical context for our melanoma findings is the comparison with the study by Weber et al. (12), who demonstrated a significant decrease in melanoma growth after 91 days of treatment with a 1% BA cream. The different outcomes are best understood by their differing objectives. The Weber et al. (12) study was an explanatory trial, designed to prove the treatment’s efficacy and under ideal circumstance, a controlled research environment with a homogeneous population, small tumors, and unblinded investigators. Our study, in contrast, was a pragmatic study designed to assess the treatment’s effectiveness in a real-world setting, with a heterogeneous population of client-owned animals and the inherent variabilities of owner compliance. The absence of a significant effect at 30 days in our study does not contradict their findings but rather provides a crucial timeline. It strongly suggests the therapeutic effect of topical betulinic acid is not apparent within the first month of use under typical clinical conditions, a vital piece of information for managing the expectations of veterinarians and horse owners.

An unexpected finding was the considerable tumor regression observed in the placebo groups for both diseases, particularly the 71% of placebo-treated melanomas that decreased in volume. This raises several possibilities. First, the placebo cream base itself may have therapeutic properties. The vehicle contained medium-chain triglycerides (MCTs), which have been suggested to have anti-neoplastic effects, potentially via inhibition of inflammation (17). Second, the physical act of massaging a cream onto the tumors may have stimulated a local immune response. Finally, for the sarcoid group, spontaneous regression is a known, if unpredictable, phenomenon that may have confounded the results (16).

The pragmatic design of this study carries both strengths and limitations. The primary limitation is the potential for inconsistent owner adherence to the protocol, and the use of multiple veterinarians for follow-up measurements may have introduced variability. However, these are common and accepted features of clinically relevant study. Another potential limitation are differences in biological behavior between melanoma groups could have influenced outcomes. With that considered, randomization should theoretically distribute tumor types and grades evenly between groups. The chief strength is the study’s high external validity, or generalizability. The results are directly applicable to the diverse population of horses that veterinarians treat daily. A notable ancillary finding was that pre-treatment biopsy of sarcoid tumors did not result in lesion deterioration, which supports other recent work challenging this long-held belief (18).

In conclusion, this study demonstrates that while topical betulinic acid is safe, a 30-day treatment interval is insufficient to determine its effectiveness for equine melanoma and sarcoid under real-world conditions. The 30-day trial period was initially selected based on available safety data, expected time to observe early anti-proliferative effects, and ethical considerations regarding prolonged placebo exposure (particularly for sarcoids). For veterinarians, the key takeaway is that treatment courses longer than 30 days are necessary, and clients should be advised not to expect rapid results. Future studies should focus on a longer treatment interval (≥90 days) and consider a control group using a cream base without MCTs to isolate the effects of the active compound. Additionally, Future studies with larger sample sizes may allow stratification by tumor size to better assess size-dependent treatment response.

## Data Availability

The raw data supporting the conclusions of this article will be made available by the authors, without undue reservation.
